# Spinal Multimorbidity, Sex-Associated Disability, and Consultation Patterns in Octogenarians and Nonagenarians: A 30-Year Single-Center Retrospective Study

**DOI:** 10.3390/jpm16100508

**Published:** 2026-09-29

**Authors:** Gabriel Oswaldo Alonso Cuéllar, Kai-Uwe Lewandrowski, Jorge Alberto Pérez Terrazas, Carolina Ramírez Martínez, Viviana Marcela Plazas Bedoya, Juan Sebastián Manchola Gómez, José Gabriel Rugeles Ortiz, Nicolás Prada Ramírez, Jorge Felipe Ramírez León

**Affiliations:** 1 Latin American Endoscopic Spine Surgeons LESS Invasiva Academy, Bogotá, D.C. 110111, Colombia; 2 Grupo de Investigación en Ortopedia y Traumatología, Departamento de Ortopedia y Traumatología, Clinica Colsanitas S.A., Bogotá, D.C. 111321, Colombia; 3 Fundación Universitaria Sanitas, Bogotá, D.C. 110311, Colombia; 4 Center for Advanced Spine Care of Southern Arizona, Tucson, AZ 85712, USA; 5 Clínica Reina Sofia Pediátrica y de la Mujer, Departamento de Ortopedia y Traumatología, Clinica Colsanitas S.A., Bogotá, D.C. 110121, Colombia; 6 Clínica Foscal Internacional, Bucaramanga 681004, Colombia

**Keywords:** low back pain, aged 80 and over, octogenarians, nonagenarians, spinal multimorbidity, Oswestry Disability Index, sex differences, referral patterns

## Abstract

**Purpose**: To characterize baseline spinal multimorbidity, we investigated sex-associated differences in disability, and the temporal distribution of patients aged 80 years or older presenting to a tertiary spine center over three decades. **Methods**: We performed a retrospective single-center study (1994–2024). Of 3199 screened unique patient medical records evaluated for low back pain, 193 unique patients were age-eligible; 71 were excluded for prespecified record-quality criteria, leaving 122 patients in the analytical cohort. Baseline Visual Analog Scale (VAS) scores were available for all 122 patients and Oswestry Disability Index (ODI) scores for 99. Diagnoses required clinical-imaging concordance. A Lumbar Pathology Count (LPC) summarized seven coexisting degenerative diagnosis categories. Temporal distributions across study periods (D1: 1994–2004, D2: 2005–2014, D3: 2015–2024) were examined descriptively. Multivariable robust linear regression evaluated the association between sex and ODI. **Results**: Mean age was 84.2 years, and median VAS was 8/10. Among 99 patients with ODI data, mean ODI was 47.6%. Women with complete sex and ODI data had higher disability than men (51.5% vs. 39.8%; *p* = 0.002; Cohen d = 0.66). In the adjusted model (n = 98), female sex remained associated with a 12.3-point higher ODI (95% CI 4.5–20.2; *p* = 0.002). Most patients (76.2%) had more than one lumbar diagnosis (mean LPC, 1.99), but LPC was not significantly correlated with VAS or ODI. From D2 to D3, facet arthrosis increased from 31.3% to 65.3% and central disc herniation decreased from 37.5% to 9.9%; both remained significant after Benjamini–Hochberg correction (q = 0.039). The analytical cohort comprised five patients in D1, 16 in D2, and 101 in D3. **Conclusions**: In this selected referral-center cohort, advanced-age patients presented with severe baseline pain, disability, and frequent coexisting lumbar degeneration. Female sex was independently associated with higher baseline disability. Temporal patient distributions reflect complete-record inclusion criteria rather than population epidemiology. These findings emphasize the necessity of explicit functional phenotyping in late-life spine care.

## 1. Introduction

The global increase in life expectancy has created a widening need to distinguish lifespan from healthspan, particularly as chronic musculoskeletal disorders accumulate in late life [1,2,3]. Degenerative spinal disease and low back pain (LBP) are prominent contributors to disability in older adults, and octogenarians and nonagenarians increasingly undergo specialist evaluation and, when appropriate, surgical treatment [4,5,6,7,8]. Middle-income countries such as Colombia are experiencing an accelerated demographic transition characterized by rapid expansion of the population aged 80 years or older [9].

LBP in extreme longevity is clinically important because pain-related mobility restriction can interact with frailty, osteopenia, sarcopenia, social isolation, and cognitive vulnerability [10,11,12,13,14,15,16]. These complex interactions do not imply that imaging degeneration alone explains disability. Rather, the aging spine should be evaluated within the broader clinical context of function, symptoms, and physiologic reserve [17,18,19,20]. Global Burden of Disease projections further suggest that the absolute disability burden from musculoskeletal disorders will continue to rise as populations age [21].

Long-term institutional data focused exclusively on patients aged 80 years or older remain limited, especially in middle-income settings. The present study therefore had three objectives: (1) to describe baseline pain, functional disability, and coexisting lumbar degenerative diagnoses in octogenarians and nonagenarians presenting to a tertiary spine referral center; (2) to examine whether baseline disability differed by recorded biological sex after adjustment for major measured covariates; and (3) to characterize the temporal distribution of analytically eligible oldest-old patients across three study periods spanning 30 years (1994–2004, 2005–2014, and 2015–2024). We hypothesized that baseline symptom severity and age at presentation would remain stable across observational periods, and evaluated sex-associated disability as a prespecified observational comparison.

## 2. Materials and Methods

### 2.1. Study Design and Patient Selection

We performed a 30-year (1994–2024) retrospective observational study at a tertiary spine referral center. Reporting was structured according to the Strengthening the Reporting of Observational Studies in Epidemiology (STROBE) recommendations [22]. For this study, the oldest-old population was operationally defined as patients aged 80 years or older, consistent with prior spine studies focused on octogenarians and nonagenarians [4,5,6].

A total of 3199 unique patient medical records (each representing an individual patient at initial LBP consultation) were screened. Of these, 193 unique patients were aged 80 years or older and were assessed for record-quality criteria. Seventy-one were excluded: 51 for incomplete clinical records, 7 for absence of MRI confirmation, and 13 for absence of a baseline pain/disability assessment. The final analytical cohort comprised 122 patients. Baseline VAS was available for all 122 patients; baseline ODI was available for 99 patients (sex-specific missingness: 9/73 women [12.3%] and 14/48 men [29.2%]) and was analyzed using available-case denominators. Recorded sex was available for 121 patients. The timing of the 71 quality exclusions by study period could not be reconstructed from the archived data and is addressed as a limitation.

Pain intensity was measured on a 0–10 Visual Analog Scale (VAS), with 0 representing no pain and 10 the greatest pain intensity, and functional disability was measured with the Oswestry Disability Index (ODI), reported on the standard 0–100% scale [23,24]. Sex was recorded in the source dataset as male or female; the manuscript therefore uses the term sex rather than gender.

Lumbar diagnoses were established by correlating symptoms and physical findings with imaging. Imaging abnormalities were not intended to be classified as symptomatic pain generators solely because they were visible radiographically. A neuroradiologist and a spine surgeon reviewed the diagnostic process and resolved discrepancies by consensus. Independent reader-level ratings were not retained, so a study-specific kappa statistic could not be reconstructed. MRI and radiographic studies were acquired over three decades on multiple systems; manufacturer, model, field strength, and acquisition metadata were not harmonized across the entire period. MRI-based evaluation of foraminal stenosis and facet arthropathy followed established radiologic principles [25,26].

The cohort was stratified into D1 (1994–2004), D2 (2005–2014), and D3 (2015–2024). A Lumbar Pathology Count (LPC) was calculated as an unweighted count of seven diagnosis categories: degenerative disc disease, foraminal disc herniation, central disc herniation, central stenosis, foraminal stenosis, facet arthrosis, and facet cyst. Each category contributed a maximum of one point per patient regardless of the number of affected levels; therefore, the LPC ranged from 1 to 7 conceptually and from 1 to 4 in the observed cohort. Scoliosis and segmental instability were analyzed separately and were not included in the LPC.

### 2.2. Statistical Analysis

Normality was assessed with the Shapiro–Wilk test. Age (W = 0.916, *p* < 0.001) and VAS (W = 0.905, *p* < 0.001) were non-normally distributed and are summarized as median and interquartile range (IQR); ODI did not significantly deviate from normality among the 99 available observations (W = 0.984, *p* = 0.278) and is summarized as mean and standard deviation (SD). Because D1 contained only five patients, it was retained for descriptive context but excluded from primary decade-to-decade inferential comparisons. D2 and D3 were compared with the Mann–Whitney U test for age and VAS, an independent-samples *t* test for ODI after Levene testing, and Fisher exact tests for categorical variables when appropriate.

Pathology prevalence was calculated using within-period denominators. Six prespecified D2-versus-D3 diagnostic comparisons were evaluated with Fisher’s exact tests, applying the Benjamini–Hochberg procedure to control the false discovery rate (reported as q values). The single facet cyst case was described narratively. Associations between LPC and symptom scales (VAS and ODI) were evaluated using Spearman rank correlation. Minimum detectable absolute correlation coefficients at 80% statistical power (alpha = 0.05) were calculated using Fisher’s z transformation.

The unadjusted sex–ODI comparison used an independent-samples *t* test and Cohen’s d. A multivariable ordinary least-squares model with HC3 heteroskedasticity-consistent robust standard errors evaluated baseline ODI as the dependent variable. Prespecified covariates were female sex (male reference), age, study period coded ordinally from D1 to D3, any documented nonspinal comorbidity, prior spine surgery, and LPC. The comorbidity indicator was derived from named diagnoses in the available clinical fields and was not a validated comorbidity index. A sensitivity model additionally included baseline VAS. Available-case analysis yielded n = 98 for the adjusted models.

Temporal distributions across study periods are presented descriptively as the number of analytically eligible patients relative to total screened records per period. Inferential temporal modeling was omitted because period-specific timestamps for excluded records were unavailable.

All revised analyses were independently reproduced in Python 3.13 using SciPy 1.17.0 and statsmodels 0.14.6. Statistical significance was set at two-sided *p* < 0.05; multiplicity-adjusted pathology results are reported as q values.

### 2.3. Ethical Considerations

The study was conducted in accordance with the Declaration of Helsinki and was approved by the institutional review board of the Fundación Universitaria Sanitas (Sanitas University Foundation; CEIFUS #2714-25). All patients signed written informed consent.

## 3. Results

### 3.1. Cohort Demographics

Of 193 age-eligible patients, 122 met the analytical criteria (Figure 1). Mean age was 84.2 ± 3.5 years (median 84; IQR 81–86; range 80–99). Six patients were aged 90 years or older. Sex was documented for 121 patients: 73 women and 48 men. Women were older than men (median 84 [IQR 82–88] vs. 83 [IQR 80–85] years; *p* = 0.018, Mann–Whitney U test; Figure 2).

### 3.2. Comorbidities and Baseline Clinical Characteristics

Comorbidity documentation reflected free-text clinical records. The most frequently documented named conditions in the full cohort were hypertension (61/122; 50.0%), heart disease (27/122; 22.1%), thyroid disorders (21/122; 17.2%), diabetes (15/122; 12.3%), dyslipidemia (12/122; 9.8%), cancer (6/122; 4.9%), arthritis (5/122; 4.1%), and osteoporosis (4/122; 3.3%). Because bone-density testing, fragility-fracture history, sarcopenia, and paraspinal muscle measurements were not systematically captured, these conditions could not be evaluated as explanatory mechanisms for disability.

Radicular lower-limb pain was the presenting symptom in 67 patients (54.9%), while 55 presented with predominantly axial low back pain. Twenty-five patients (20.5%) had a documented history of prior spine surgery, 29 (23.8%) had scoliosis, and 13 (10.7%) had segmental instability. Baseline clinical characteristics by period are summarized in Table 1. D2 and D3 did not differ significantly in age, VAS, ODI, recorded sex distribution, prior spine surgery, presenting symptom pattern, scoliosis, or segmental instability.

### 3.3. Lumbar Spinal Multimorbidity

Degenerative disc disease was documented in 72 patients (59.0%), facet arthrosis in 71 (58.2%), foraminal stenosis in 41 (33.6%), foraminal disc herniation in 29 (23.8%), central disc herniation in 18 (14.8%), central stenosis in 11 (9.0%), and facet cyst in one (0.8%). Ninety-three patients (76.2%) presented with multiple coexisting lumbar pathologies. The mean LPC was 1.99 ± 0.72 (median 2; range 1–4). The most frequent combinations were degenerative disc disease with facet arthrosis (n = 41) and foraminal stenosis with facet arthrosis (n = 21).

Evaluating diagnostic trends using within-period denominators revealed two statistically significant changes between D2 and D3 following false discovery rate control: facet arthrosis increased from 31.3% to 65.3% (*p* = 0.013; q = 0.039), whereas central disc herniation decreased from 37.5% to 9.9% (*p* = 0.009; q = 0.039). Prevalence rates for degenerative disc disease, foraminal stenosis, foraminal disc herniation, and central stenosis remained stable across periods (Table 2).

### 3.4. Pain, Disability, and Sex-Associated Differences

Baseline VAS scores (n = 122) demonstrated a median pain intensity of 8/10 (IQR 7–10). Mean baseline ODI (n = 99) was 47.6 ± 18.3% (95% CI 43.9–51.2%). Within the ODI complete-case subset, 41 patients (41.4%) presented with severe disability (ODI 41–60%), 19 (19.2%) with crippled functional status (ODI 61–80%), and two (2.0%) with complete disability (>80%).

Among patients with both recorded sex and ODI data, women (n = 64) had a higher mean ODI than men (n = 34): 51.5 ± 16.6% versus 39.8 ± 19.4% (t = 3.12, df = 96, *p* = 0.002). The standardized effect size was Cohen’s d = 0.66. With the available group sizes, the minimum detectable standardized difference at 80% power and alpha = 0.05 was approximately d = 0.60. No statistically significant sex difference in baseline VAS was detected (women median 8 [IQR 7–10] vs. men median 8 [IQR 7–10]; *p* = 0.677).

In the prespecified multivariable model (n = 98), female sex remained associated with a 12.3-point higher ODI (95% CI 4.5–20.2; *p* = 0.002) after adjustment for age, study period, any documented nonspinal comorbidity, prior spine surgery, and LPC (Table 3). In a sensitivity model that additionally adjusted for baseline VAS, the association remained significant (adjusted beta 10.4 points; 95% CI 2.8–18.1; *p* = 0.007). Missing baseline ODI data differed by recorded sex: nine of 73 women (12.3%) and 14 of 48 men (29.2%) lacked baseline ODI scores. Unadjusted and adjusted sex-associated comparisons apply to available complete cases (n = 98) and may be affected by selection.

### 3.5. Pathology Count and Symptom Burden

LPC was not significantly correlated with baseline VAS (Spearman rho = 0.099, *p* = 0.280; n = 122) or ODI (rho = −0.082, *p* = 0.417; n = 99). These null results should not be interpreted as demonstrating independence. At alpha = 0.05 and 80% power, the minimum detectable absolute correlations were approximately 0.25 for the VAS analysis and 0.28 for the ODI analysis; smaller associations therefore cannot be excluded.

### 3.6. Institutional Consultation Pattern

Descriptively, the final analytical cohort comprised five patients in D1 (1994–2004), 16 in D2 (2005–2014), and 101 in D3 (2015–2024) out of 628, 790, and 1781 screened unique patient records in those respective periods (each record representing a distinct individual patient at initial evaluation). Because period-specific exclusion data for the 71 excluded age-eligible records could not be reconstructed, these figures represent the proportion of screened records meeting complete analytical inclusion criteria rather than the true proportion of all consultations involving patients aged 80 years or older. They should not be used to infer epidemiological trends or changing institutional consultation rates.

Age at consultation remained similar between the two inferentially compared periods: median 83 years (IQR 81.8–86) in D2 and 84 years (IQR 81–87) in D3 (*p* = 0.786). D1 is displayed for descriptive context only (Figure 3).

## 4. Discussion

This 30-year single-center study shows that patients aged 80 years or older who reached specialty spine evaluation presented with high baseline pain, substantial functional disability, and frequent coexisting lumbar degenerative diagnoses. Three primary observations emerge from these data. First, advanced-age patients presenting for specialist spine evaluation exhibit severe baseline pain and functional disability that remain stable across observational periods. Second, female sex is independently associated with substantially higher baseline functional disability after controlling for age, comorbidity, prior surgery, and structural pathology burden. Third, lumbar degenerative conditions in extreme longevity present predominantly as a multimorbid complex rather than isolated structural lesions.

### 4.1. Institutional Consultation Pattern and Demographic Context

The temporal distribution of analytically eligible patients across study periods (five cases in D1, 16 in D2, and 101 in D3) aligns with broader demographic aging trends in Colombia [9]. However, this temporal pattern cannot be interpreted as a true population incidence or changing healthcare utilization rate. Because period-specific timestamps for the 71 excluded age-eligible records were unavailable, the higher patient volume in recent years reflects a combination of institutional growth, evolving electronic health record archiving, increased access to high-resolution MRI, and shifting referral patterns rather than an epidemiological transition alone.

### 4.2. Diagnostic Composition and Imaging Era Effects

Using within-period denominators changed the interpretation of several diagnostic trends. Facet arthrosis increased between D2 and D3, whereas central disc herniation decreased; the remaining major diagnosis categories did not show statistically significant D2-to-D3 differences after false-discovery-rate correction. The small D1 denominator precludes reliable inference from its apparently large percentages.

The increase in documented facet disease may partly reflect improved imaging recognition rather than true biologic change. In a cadaveric series, Eubanks et al. found facet arthrosis in 100% of specimens older than 60 years when any lumbar level was considered, while L4–L5 had the highest level-specific prevalence overall (79%), not a literal 100% prevalence at L4–L5 [27]. Community-based CT data also show that facet osteoarthritis is common with aging but is not necessarily associated with LBP [28]. These observations reinforce an important distinction for the present study: degenerative imaging findings may coexist with symptoms without being the sole source of pain. Facet disease may still be clinically relevant in selected patients, including as a potential contributor to persistent symptoms after minimally invasive discectomy [29], but causal attribution requires clinical-imaging concordance.

### 4.3. Baseline Symptom Severity and Lumbar Pathology Count

Median VAS remained 8/10 and mean ODI remained in a high disability range, with no significant difference between D2 and D3. The LPC was designed only as a transparent count of coexisting diagnosis categories, not as a validated weighted index. Its lack of statistically significant correlation with the VAS or ODI does not establish that structural burden is irrelevant to symptoms. The study was powered to detect only moderate correlations (approximately |rho| >= 0.25–0.28), so small associations remain possible. In this cohort, the number of coexisting lumbar degenerative diagnoses was not significantly correlated with baseline pain intensity or functional disability.

### 4.4. Sex-Associated Disability

Women had a higher baseline ODI than men, and this association remained significant after adjustment for age, period, documented comorbidity, prior spine surgery, and LPC. The adjusted difference of approximately 12 ODI points is clinically notable, but it should be interpreted as a sex-associated baseline disparity rather than evidence of a causal sex mechanism. Importantly, the VAS comparison did not show statistical equivalence; it only showed that no significant sex difference in pain intensity was detected.

Several plausible mechanisms deserve prospective study rather than retrospective attribution. Postmenopausal bone loss, fragility fractures, paraspinal muscle loss, sarcopenia, pelvic and spinopelvic biomechanics, and sex-related differences in pain processing or social and caregiver roles could influence disability [30,31,32,33]. In the present dataset, osteoporosis was infrequently documented, and bone density, vertebral or pelvic fracture burden, paraspinal muscle morphology, frailty, caregiver status, and domestic workload were not systematically measured. These factors therefore remain unmeasured confounders and hypotheses for future work, not explanations established by this study.

### 4.5. International Registry Context and Clinical Relevance

Large international registries provide useful context for the clinical severity represented by older spine patients but address a different question from the present baseline study. In the Norwegian Registry for Spine Surgery, 178 patients aged 80 years or older undergoing decompression for lumbar stenosis were analyzed within a 1503-patient multicenter cohort and experienced meaningful one-year improvement [34]. Spine Tango analyses of 1764 and 4768 patients found that older age altered parts of the complication profile but did not eliminate clinically important benefits after treatment for lumbar stenosis [35,36]. Similarly, the U.S. N2QOD registry included 4370 lumbar operations, with 1020 patients aged 70 years or older, and demonstrated substantial patient-reported benefits in the older group [37].

Those registry studies are treatment cohorts and should not be used to infer treatment effectiveness from our baseline data. Their relevance here is that advanced age does not define a homogeneous clinical phenotype: older patients present with varied comorbidity, pathology, and functional reserve. Geroscience and mobility frameworks therefore support measuring function explicitly rather than relying on chronological age or a single structural lesion alone [38,39,40]. In this cohort, the practical implication is straightforward: comprehensive baseline assessment is justified, but the current analysis does not establish that any particular treatment strategy improves outcomes.

This same institutional population has been reported separately for treatment and follow-up outcomes, including postoperative VAS, ODI, modified Macnab outcomes, complications, and reinterventions [41]. To avoid duplicate outcome reporting, the present manuscript is intentionally restricted to baseline phenotype, sex-associated disability, diagnostic composition, and institutional consultation patterns.

### 4.6. Limitations

Several limitations should be emphasized. First, this was a retrospective, single-center referral cohort and is vulnerable to referral and selection bias. Of 193 age-eligible patients, 71 were excluded for record-quality criteria; the distribution of those exclusions by decade was unavailable, so era-dependent differences in record completeness cannot be quantified. Second, institutional growth, changing referral patterns, improved record archiving, and broader imaging availability across decades may explain the larger number of eligible patient records retrieved in recent periods. Because period-specific timestamps for excluded records were unavailable, temporal patient counts reflect complete-record availability rather than true population epidemiology or changing healthcare utilization rates.

Third, MRI and radiographic acquisition occurred across multiple systems over 30 years, and device-specific technical metadata could not be harmonized retrospectively. Fourth, diagnoses were consensus-adjudicated clinically and radiographically, but independent reader-level ratings were not preserved; consequently, study-specific inter-rater reliability statistics cannot be reported. Fifth, ODI was missing in 23 of 122 patients and sex in one patient; analyses therefore used explicit available-case denominators, and missingness may not be random.

Sixth, comorbidities were retrospectively documented in heterogeneous clinical fields and were not captured with a validated comorbidity index. Important potential confounders—including bone mineral density, fragility fractures, sarcopenia or paraspinal muscle atrophy, pelvic parameters, frailty, socioeconomic status, caregiver dynamics, and psychosocial factors—were not measured systematically. The multivariable model reduces but cannot eliminate residual confounding. Seventh, D1 contained only five patients and was therefore descriptive. Finally, the present manuscript does not analyze treatment effectiveness or longitudinal functional decline; those endpoints cannot be inferred from baseline VAS and ODI values.

## 5. Conclusions

In this selected single-center cohort of patients aged 80 years or older, specialty spine consultation was characterized by high baseline pain, substantial disability, and frequent coexisting lumbar degenerative diagnoses. Female sex was associated with a higher baseline ODI after adjustment for measured covariates. The number of coexisting lumbar diagnoses was not demonstrably correlated with the VAS or ODI, although small associations cannot be excluded. Temporal patient distributions across study periods reflect complete-record inclusion criteria rather than true population epidemiology or institutional consultation trends. These results support explicit functional assessment and careful clinical-imaging correlation in the oldest-old and provide a rationale for prospective multicenter studies of mechanisms, treatment selection, and longitudinal outcomes.

## Figures and Tables

**Figure 1:**
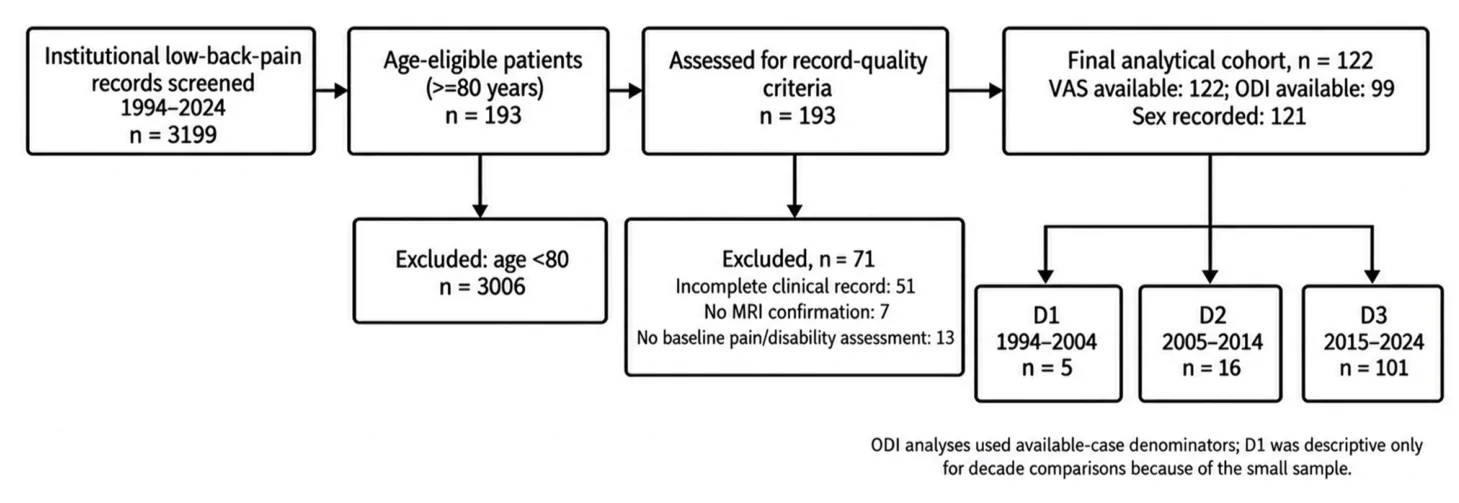
Study flow. Of 3199 institutional LBP records screened, 193 patients were aged 80 years or older. After 71 record-quality exclusions, 122 patients formed the analytical cohort. VAS was available for all 122 patients, ODI for 99, and recorded sex for 121. Exclusion counts by study period were not recoverable. D1: 1994–2004; D2: 2005–2014; D3: 2015–2024; VAS: Visual Analog Scale; ODI: Oswestry Disability Index; MRI: magnetic resonance imaging.

**Figure 2:**
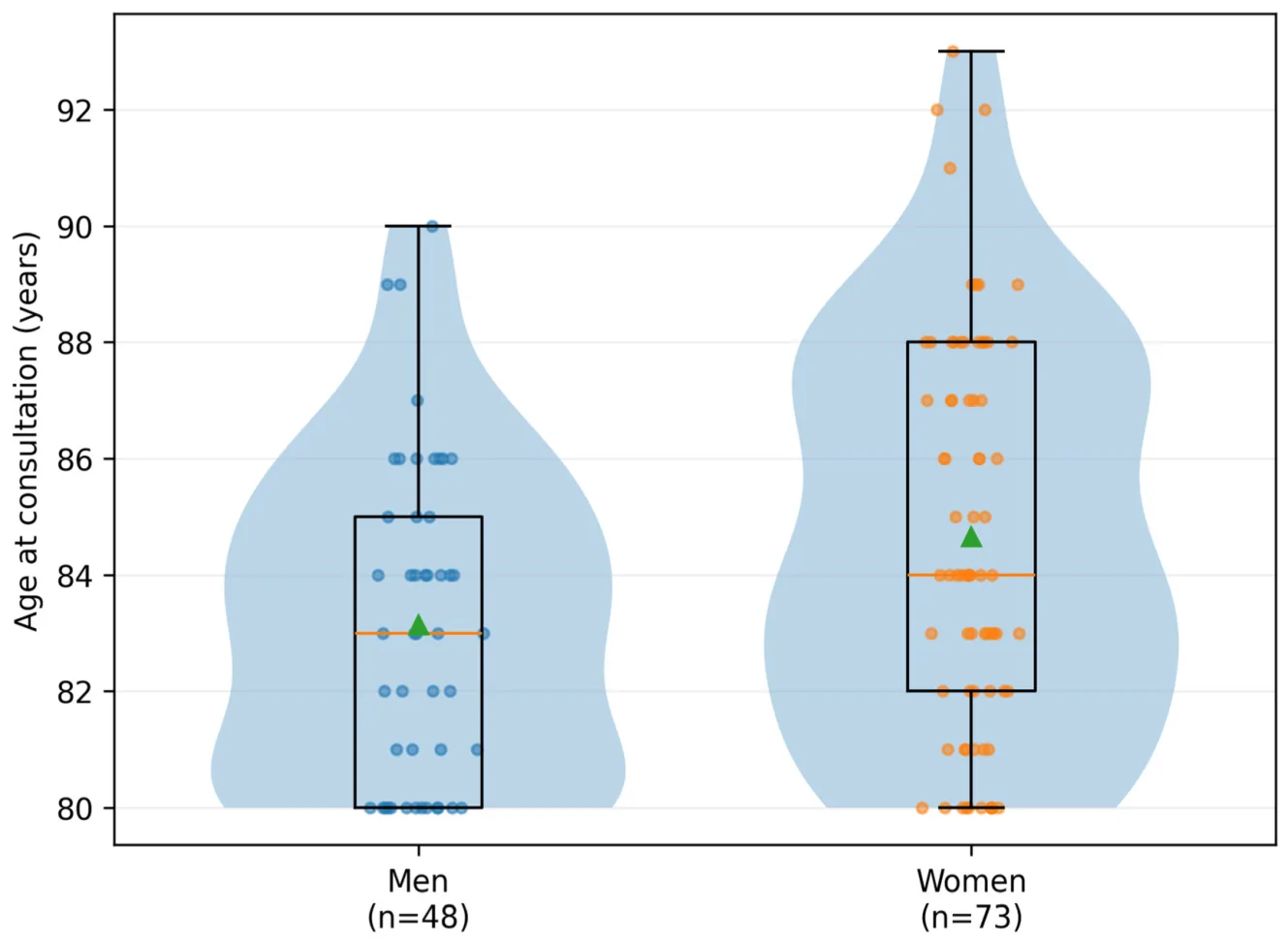
**Age distribution by recorded sex.** The plot includes 73 women and 48 men; one patient with missing sex was not plotted. Women were older at consultation than men (median 84 [IQR 82–88] vs. 83 [IQR 80–85] years; *p* = 0.018, Mann-Whitney U test). Blue and orange dots represent individual observations for men and women, respectively; green triangles indicate group means; horizontal orange lines inside boxplots represent medians. Violin distributions and boxplots illustrate sample density and spread.

**Figure 3:**
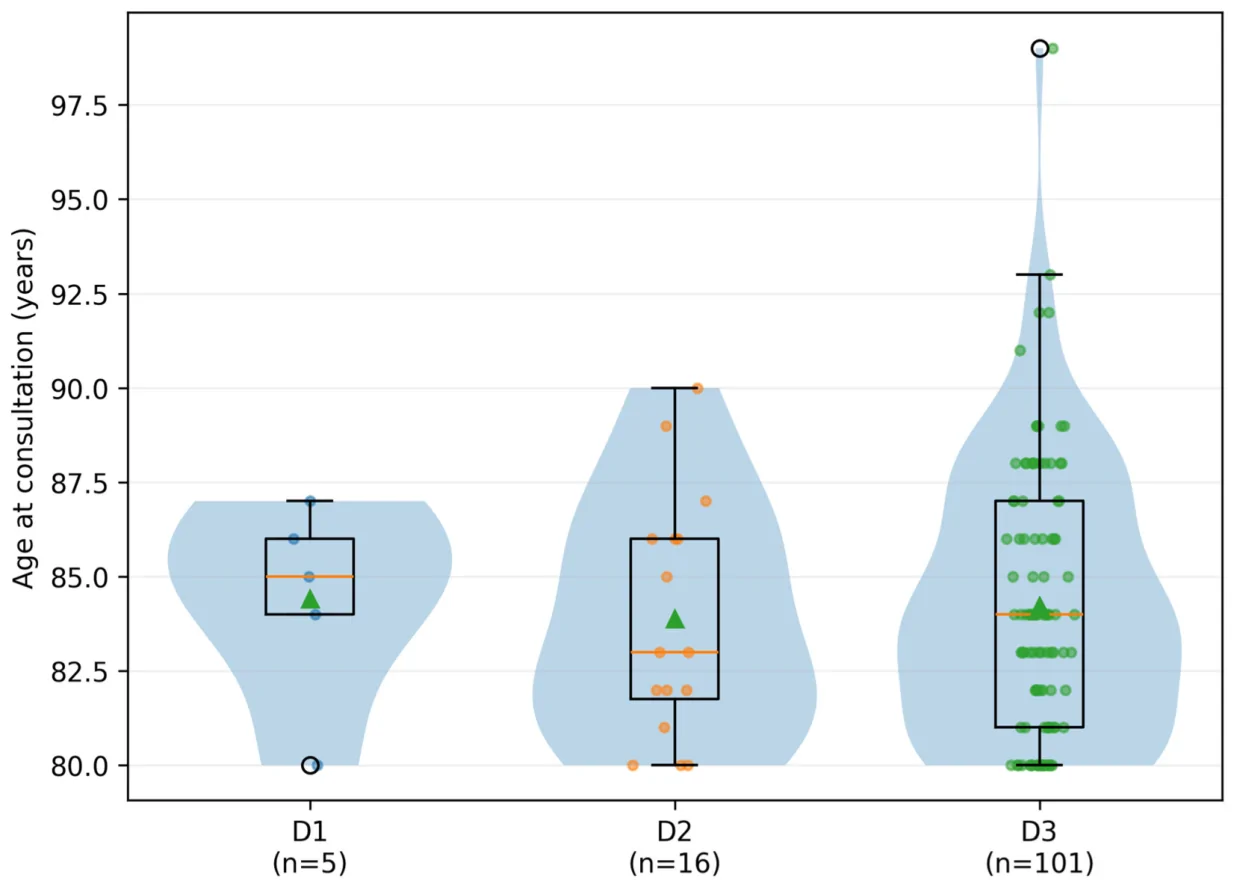
**Age distribution by study period.** D1 (n = 5) is displayed descriptively because of the small sample. Median age did not differ between D2 and D3 (83 [IQR 81.8–86] vs. 84 [IQR 81–87] years; *p* = 0.786, Mann-Whitney U test). The maximum observed age was 87 years in D1, 90 years in D2, and 99 years in D3. Blue, orange, and green dots represent individual patient observations for D1, D2, and D3, respectively; green triangles indicate group means; horizontal orange lines inside boxplots represent medians. Violin plots and boxplots illustrate sample density and spread.

**Table 1:** Demographic and baseline clinical characteristics by study period.

Characteristic	D1 1994–2004 (n = 5)	D2 2005–2014 (n = 16)	D3 2015–2024 (n = 101)	D2 vs. D3 *p* Value
Female sex, n/N (%)	4/5 (80.0)	12/16 (75.0)	57/100 (57.0)	0.272
Age, median (IQR), years	85 (84–86)	83 (81.8–86)	84 (81–87)	0.786
Prior spine surgery, n/N (%)	1/5 (20.0)	4/16 (25.0)	20/101 (19.8)	0.739
Radicular presentation, n/N (%)	3/5 (60.0)	9/16 (56.3)	55/101 (54.5)	1.000
Scoliosis, n/N (%)	2/5 (40.0)	4/16 (25.0)	23/101 (22.8)	1.000
Segmental instability, n/N (%)	0/5 (0)	3/16 (18.8)	10/101 (9.9)	0.384
VAS, median (IQR)	7 (7–8)	8 (7.8–10)	8 (7–10)	0.544
ODI, mean ± SD, % (n)	60.4 ± 15.1 (5)	46.0 ± 14.2 (13)	47.0 ± 18.9 (81)	0.854

Note: D1 is shown descriptively; *p* values compare D2 with D3 only. Fisher exact tests were used for categorical variables, Mann–Whitney U tests for age and VAS, and an independent-samples *t* test for ODI. One D3 patient had missing sex. ODI was available for 5/5 D1, 13/16 D2, and 81/101 D3 patients. VAS = Visual Analog Scale; ODI = Oswestry Disability Index; IQR = interquartile range; SD = standard deviation. The marked imbalance in period-specific sample sizes may reflect institutional growth, changing referral patterns, record availability, and imaging access and should not be interpreted as a population-level epidemiologic trend.

**Table 2:** Lumbar degenerative diagnoses using within-period denominators.

Diagnosis	Overall n (%)	D1 n/N (%)	D2 n/N (%)	D3 n/N (%)	D2 vs. D3 *p*	BH q
Degenerative disc disease	72 (59.0)	2/5 (40.0)	11/16 (68.8)	59/101 (58.4)	0.585	0.702
Facet arthrosis	71 (58.2)	0/5 (0)	5/16 (31.3)	66/101 (65.3)	0.013	0.039
Foraminal stenosis	41 (33.6)	0/5 (0)	7/16 (43.8)	34/101 (33.7)	0.574	0.702
Foraminal disc herniation	29 (23.8)	3/5 (60.0)	1/16 (6.3)	25/101 (24.8)	0.117	0.235
Central disc herniation	18 (14.8)	2/5 (40.0)	6/16 (37.5)	10/101 (9.9)	0.009	0.039
Central stenosis	11 (9.0)	1/5 (20.0)	1/16 (6.3)	9/101 (8.9)	1.000	1.000
Facet cyst	1 (0.8)	0/5 (0)	0/16 (0)	1/101 (1.0)	—	—

Note: Patients may contribute to more than one diagnosis row. Each diagnosis category contributed a maximum of one point to the LPC regardless of the number of involved levels. *p* values are Fisher exact tests comparing D2 and D3. Benjamini–Hochberg (BH) q-values control the false discovery rate across the six prespecified pathology comparisons; the single facet-cyst case was descriptive only. LPC = Lumbar Pathology Count. The marked imbalance in period-specific sample sizes may reflect institutional growth, changing referral patterns, record availability, and imaging access and should not be interpreted as a population-level epidemiologic trend.

**Table 3:** Multivariable robust linear regression for baseline ODI (n = 98).

Predictor	Adjusted Beta (ODI Points)	95% CI	*p* Value
Female sex (vs. male)	12.31	4.45 to 20.16	0.002
Age, per year	0.18	−0.94 to 1.30	0.758
Study period, per period	−3.79	−10.17 to 2.58	0.244
Any documented nonspinal comorbidity	6.95	−0.65 to 14.55	0.073
Prior spine surgery	9.29	−1.52 to 20.11	0.092
Lumbar Pathology Count, per diagnosis	−2.35	−7.68 to 2.99	0.389

Note: Ordinary least-squares regression with HC3 robust standard errors. Study period was coded D1 = 1, D2 = 2, D3 = 3 (ordinal). The model R^2^ was 0.163. The comorbidity variable indicates at least one named nonspinal comorbidity in the available clinical fields and is not a validated comorbidity index. ODI = Oswestry Disability Index; CI = confidence interval.

## Data Availability

The data supporting the findings of this study are available from the corresponding author upon reasonable request, subject to institutional and ethical restrictions.
